# Gender Differences in the Self-Reporting of Adult Attachment Style and Psychological Symptoms

**DOI:** 10.1192/bjo.2026.11105

**Published:** 2026-06-30

**Authors:** Daniel Napack, Kristina Burns, Emily Kim

**Affiliations:** 1 Northwell Health, Port Jefferson, USA; 2 New York Institute of Technology, Old Westbury, USA

## Abstract

**Aims:**

Gender differences are hypothesized to influence the reporting of attachment styles and psychological symptoms. Culturally, masculine characteristics are often linked with physical and aggressive tendencies, while feminine characteristics are associated with internalizing problems such as depression and anxiety. Some theoretical perspectives suggest that attachment styles may reflect different gender-specific mating strategies. This study investigated gender differences in self-reported adult attachment and psychological symptoms within a sample of patients from a cardiology outpatient clinic.

**Methods:**

A total of 186 patients awaiting consultation at a private outpatient cardiology clinic completed a packet of self-reports. The sample comprised 55.7% males (one participant did not report gender) and 85.5% Caucasian individuals, with an average age of 66.2 years. We analysed gender differences in adult attachment using the 12-item Experiences in Close Relationship-Short Form (ECR-SF) and scores on the SPECTRA: Indices of Psychopathology, a broadband self-report measure of psychopathology.

**Results:**

Males scored significantly higher than females on several measures. Specifically, males reported higher ECR-SF attachment anxiety [t(139)=2.12, p=0.03], and higher scoreson SPECTRA’s severe aggression scale [t(120)=2.21, p=0.03], antisocial tendencies [t(120)=2.51, p=0.02], externalizing spectrum [t(120)=2.48, p=0.01], grandiose ideation [t(119)=2.93, p<0.01], and reality impairing spectrum [t(119)=3.00, p<0.01].

**Conclusion:**

Our findings indicate that males reported higher scores on several psychological symptom scales, many of which align with theoretically externalizing strategies (e.g. aggression). More surprising findings included higher male scores on grandiose ideation and the reality impairing spectrum of the SPECTRA. Additionally, males exhibited higher attachment anxiety, which is unexpected given that females typically score higher on this dimension in most samples, with the exception of some Asian populations. Future research is warranted to further investigate these findings and enhance our understanding of gender differences in self-reports.

